# Sediment temperature characteristics and their relation to distribution patterns of two sentinel crab species in intertidal flats in western Japan

**DOI:** 10.1038/s41598-024-51515-8

**Published:** 2024-01-09

**Authors:** Akihiko Koyama, Ryutei Inui

**Affiliations:** 1 Fishery Research Laboratory, Kyushu University, Fukutsu, Fukuoka Japan; 2 Faculty of Socio-Environmental Studies, Fukuoka Institue of Technology, Fukuoka, Fukuoka Japan

**Keywords:** Environmental sciences, Limnology, Ocean sciences

## Abstract

In the context of global climate change, monitoring focused on temperature and benthic animals in intertidal flats and the development of metrics to assess climate change and ecosystem responses are essential for a sustainable society. However, few studies have assessed the relationship between intertidal sediment temperature and the distribution of benthic animals. To address this gap, in the present study, intertidal sediment temperatures were observed in 12 intertidal flats in 11 survey areas over 335 days, from October 2, 2019, to August 31, 2020, using water temperature data loggers. The characteristics of intertidal sediment temperatures were variable among the survey areas, and a correlation analysis suggested that such characteristics are possibly influenced by various spatial-scale factors, such as geographical, basin, and habitat scales. Furthermore, two sentinel crab species, *Macrophthalmus japonicus* and *Macrophthalmus banzai* were collected, and the number of wintering individuals of each species was estimated based on their carapace width to analyze the changes in abundances of the two species in each survey area. The results show that the number of days with daily minimum temperature ≥ 19 °C was the factor that influenced the abundance rate, suggesting that *M. japonicus* and *M. banzai* populations may decrease and increase, respectively, according to future climate change in Japan. Our findings emphasize the importance of long-term monitoring of sediment temperatures and benthic animals in intertidal flats to evaluate the influence of future climate change.

## Introduction

The management of estuarine ecosystems is an urgent global issue. Estuaries are transitional zones between terrestrial and marine areas, where various intertidal habitats such as tidal flats, salt marshes, coral reefs, and mangrove forests are formed^1,2^. These intertidal habitats provide several ecosystem services; nevertheless, they are threatened by degradation and loss due to anthropogenic pressures^3,4^. In recent years, climate change, which is one of the anthropogenic impacts, has been suggested to have caused the expansion of the distribution range of estuarine/marine species^5,6^ and a decline in the populations of intertidal benthos^7^. Consequently, there are concerns regarding the negative impacts for the existing estuarine ecosystems^8^. Therefore, monitoring focused on temperature and intertidal benthos, and the development of metrics to assess climate change and ecosystem responses are essential for a sustainable society.

Temperature trends differ between the surface and sediments of intertidal flats^9^. Intertidal flats are sedimentary landscapes that are formed in the lower intertidal zones^10^, and are routinely flooded and dried out according to tidal cycles. Temperature fluctuations in intertidal sediments are smaller than those in the surface^9,11^. The intertidal sediment temperature is affected by tides, solar radiation, and groundwater from land areas^12–14^. Although several studies have assessed the trends in sediment temperature, few have assessed the relationship between temperature and benthic animals^15^. In particular, infaunal benthos have a life history dependent on intertidal sediments and further research is needed to address this gap.

Our study focused on two species of sentinel crabs *Macrophthalmus japonicus* and *Macrophthalmus banzai*, which are infaunal brachyuran decapods that dig burrows in sandy or muddy intertidal flats^16^. These sentinel crabs affect intertidal flat ecosystems through bioturbation^17,18^, and have been utilized as effective indicators to assess environmental changes in intertidal zones^19,20^. Thus, studies on these two species are expected to contribute to the conservation of intertidal flat ecosystems and the establishment of monitoring techniques.

Despite similar habitat requirements, *M. japonicus* and *M. banzai* are considered to have adapted to cool and warm water, respectively, because the distribution range of *M. japonicus* is between temperate and subarctic zones, and *M. banzai* is distributed in lower latitude areas than *M. japonicus*^21^. Before the 1980s, two species were distributed independently or coexisted in western Japan^16,22^, whereas only *M. japonicus* was distributed in eastern Japan^21^. In recent years, however, the distribution and wintering of *M. banzai* has been confirmed in eastern Japan^23,24^. Although it is possible that the composition ratio of the two species may change in response to climate change in western Japan, where they coexist, no study has evaluated the relationship between the distribution patterns of these two species and intertidal sediment temperatures.

To address this gap, the objective of the present study was to monitor intertidal sediment temperatures and investigate the distribution of these two species in western Japan and their relationships with intertidal sediment temperature. Winter temperatures have been shown to affect the survival and distribution expansion of brachyuran crab larvae and juveniles^25–27^. Based on the information that *M. japonicus and M. banzai* are dormant in the intertidal sediment during winter^16^, we hypothesized that the spatial distribution patterns of these two species are closely related to the intertidal sediment temperature during that season.

## Material & methods

### Data collection

**Figure 1:**
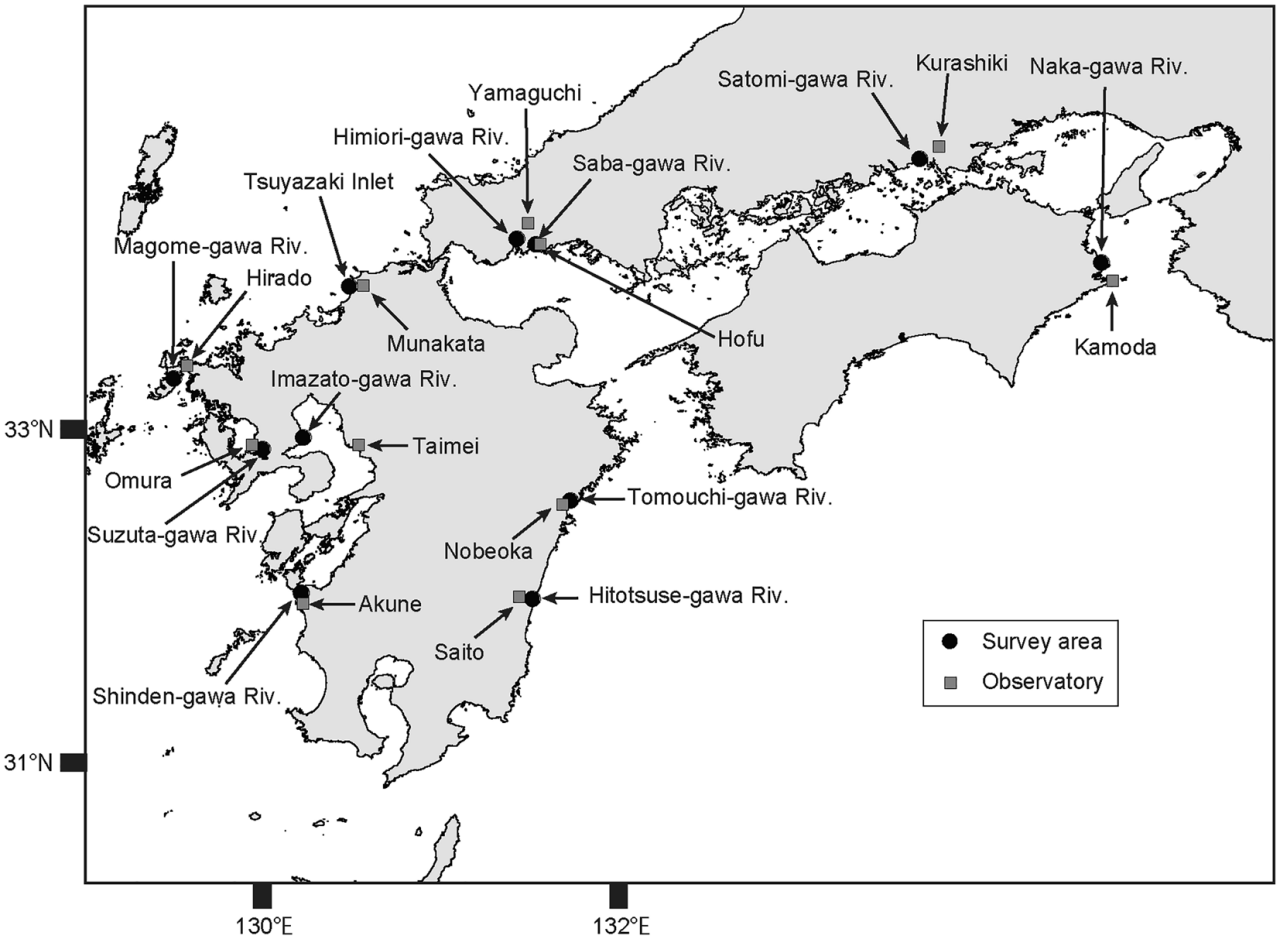
Locations of survey areas and observatory stations.

Eleven intertidal flats were selected as survey areas in western Honshu, Shikoku, and Kyushu, western Japan (Fig. 1). These sites represent the geographical distributional ranges where *M. japonicus* and *M. banzai* are sympatrically distributed^21^, and the presence of *M. japonicus* and/or *M. banzai* has been found in previous surveys^2,28,29^. In Japan, these two species inhabit sandy and muddy intertidal flats in the lower intertidal zone and are active on the tidal flat surface during daytime low tides in the non-winter months^16,22^. The first survey was conducted between July and October 2019 and the second survey was conducted between September and November 2020 during daytime low tide (Table 1).

**Table 1:** Location and basin area in each survey area.

Survey area name	Date (YYYY/MM/DD)		Basin area (km^2^)
	Set	Collection	
Naka-gawa Riv	2019/10/02	2020/09/19	874
Satomi-gawa Riv	2019/08/14	2020/09/18	81
Saba-gawa Riv	2019/08/13	2020/09/14	460
Himiori-gawa Riv	2019/08/13	2020/09/14	322
Tsuyazaki Inlet	2019/07/16	2020/09/27	NA
Magome-gawa Riv	2019/08/25	2020/11/02	105
Suzuta-gaaw Riv	2019/08/25	2020/09/15	18
Imazato-gawa Riv	2019/08/26	2020/09/15	3
Tomouchi-gawa Riv	2019/08/13	2020/10/04	1820
Hitotsuse-gawa Riv	2019/08/29	2020/09/21	852
Shinden-gawa Riv. (H)	2019/09/16	2020/09/17	10
Shinden-gawa Riv. (L)	2019/09/16	2020/09/17	10

*NA* data not available because the Tsuyazaki Inlet is not included in the basin.

Set and collection indicate the date of first and second survey, respectively. Water temperature loggers were installed during the first survey and collected during the second survey.

(*H*) and (*L*) in Shinden-gawa Riv. indicate high-ground and a low-ground zones, respectively.

During the first survey period, the habitat range of sentinel crabs (i.e. zonation) was confirmed by visual observation in each survey area. A water temperature logger (HOBO Water Temp Pro V2 Data Logger, ONSET, Bourne, MA, USA) was set up at the ground level intermediate to the sentinel crab zonation. The sentinel crab habitat in the Shinden-gawa River was divided by a road into high- and low-ground sections, excluding the intermediate section. Consequently, two loggers were set up in this survey area: a high-ground zone (H) and a low-ground zone (L). The loggers were set up in the sediment at a depth of approximately 10–15 cm below the surface, because the burrow depth of *M. japonicus* is approximately 10–15 cm^30^. The loggers recorded temperatures at 15-min intervals and were collected during the second survey period.

Sentinel crabs were obtained during the setting up and collection of the water temperature loggers in each survey area. When *M. japonicus* and *M. banzai* coexist in the lower intertidal zone, the former inhabits the areas higher than those inhabited by the latter species in the zone^22^. In the ten survey areas, except for the Shinden-gawa River, we randomly excavated six quadrats (20 × 20 cm, at a depth of 10 cm) within a radius of 10 m of the water temperature logger. The excavated sediments were placed in a hand net with a 2 mm mesh, washed, and the remaining crabs in the hand net were collected. In the Shinden-gawa River, six quadrats were set up in the vicinity of each water temperature logger at sites H and L, respectively; a total of 12 quadrats were set up. The collected crabs were stored in ice and brought to the laboratory for species identification and measurements of maximum carapace width (CW). Ovigerous females were recorded, and females with carapace widths less than 7.0 mm were regarded as unidentified females because it was difficult to identify them as *M. japonicus* or *M. banzai*.

The number of individuals of each species with a size considered overwintering was recounted according to Henmi^16^. The recruitment of *M. japonicus* was observed in September, and its CW was less than 10 mm in December^16^. Therefore, *M. japonicus* individuals with greater than 10 mm CW were regarded as overwintering individuals. Recruitment of *M. banzai* was observed from June, and by November, the CW of the recruits and small overwintering individuals seemed to partially overlap in the range of 8–10 mm in CW^16^. Since the smallest ovigerous female was 8.2 mm in CW in the specimens of the present study (see “Results” section), *M. banzai* with greater than 8.0 mm CW were regarded as overwintering individuals.

Two surface sediment samples were collected at 8 cm diameter and 3 cm depth in each survey area at the time of setting up and collecting the water temperature logger (i.e. four samples per survey area). As with the collection of sentinel crabs, eight sediment samples were obtained from the Shinden-gawa River because two samples were collected from each of the H and L sites. The collected sediments were dried in the laboratory at 100 °C using a constant temperature drying oven (ETTAS, AS ONE, Osaka, Japan), and sediments were sieved through a sieve with 0.063 mm mesh to determine silt and clay content. The average of two samples at the time of collection of the water temperature loggers (i.e. samples collected in the second survey) was used for the analysis.

Air temperature data for the observation period were downloaded from the Japan Meteorological Agency (https://www.data.jma.go.jp/obd/stats/etrn/index.php). Hourly air temperature information was obtained from the observatories nearest to each survey area. The elevation of each observatory varied, and data were often missing (Table 2).

**Table 2:** Elevation of the nearest observatory to each survey area and the number of temperature observations during the survey period from October 2, 2019 to August 31, 2020.

Site name	Observatory name	Elevation (m)	N. of observations
Naka-gawa Riv	Kamoda	10	8032
Satomi-gawa Riv	Kurashiki	3	8038
Sabla-gawa Riv	Hofu	4	8037
Himiori-gawa Riv	Yamaguchi	18	8037
Tsuyazaki Inlet	Munakata	7	8037
Magome-gawa Riv	Hirado	58	8009
Suzuta-gaaw Riv	Omura	3	8038
Imazato-gawa Riv	Taimei	15	8035
Tomouchi-gawa Riv	Nobeoka	19	8040
Hitotsuse-gawa Riv	Saito	8	8033
Shinden-gawa Riv. (H)	Akune	40	8038
Shinden-gawa Riv. (L)	Akune	40	8038

Elevation was assessed on the basis of Tokyo Peil (T.P.)

### Statistical analysis

The dates of setting up and collection of the water temperature loggers were different for each survey area. Therefore, the temperature data in the intertidal sediment in each survey area and each observatory from October 2, 2019, to August 31, 2020 (i.e. 335 days), which is the period of overlap among the survey areas, were used for the analysis. The daily mean temperature, maximum temperature, minimum temperature, and range (range of maximum–minimum temperature) were calculated. The Friedman test was performed to determine whether there were differences in intertidal sediment temperatures between the survey areas. When significant differences were found using the Friedman test, pairwise comparison tests (Scheffe’s test) were performed. The monthly mean temperature, mean daily range, maximum temperature, and minimum temperature in each survey area were extracted to evaluate seasonal changes.

For each survey area, the mean, maximum, and minimum temperatures and the mean daily range in the intertidal sediment for the observation period (335 days) were extracted. Pearson’s correlation coefficients were calculated between these four temperature variables and the mean air temperature, mean daily range of air temperature, maximum air temperature, minimum air temperature, silt and clay content, latitude, longitude, and basin area (log transformed vale). The data for the Tsuyazaki Inlet were excluded from the correlation analysis with the basin area because this survey area was not included in the basin.

To evaluate the relationship between the intertidal sediment temperature and air temperature in each survey area, scatter plots of the daily mean intertidal sediment temperature and air temperature for 335 days were generated. The average daily air temperatures recorded from the 11 observatories were calculated and used as the standardized air temperatures.

To assess the relationship between the occurrence patterns of *M. japonicus* and *M. banzai* and environmental factors, a correlation analysis was conducted and a generalized linear model (GLM) was subsequently constructed. Because the setting up of the water temperature loggers and collection of the sentinel crabs were conducted under different conditions in the Shinden-gawa River than in the other survey areas, the average values of the abundance of each sentinel crab species and environmental factors obtained from sites H and L were calculated and used for the analysis. Spearman’s rank correlation coefficients were calculated for the number of individuals of each species, silt and clay content, latitude and longitude values, and intertidal sediment temperature. Daily maximum and minimum temperatures of the intertidal sediments were used for the analysis. Thresholds were set at 1 °C intervals for the daily maximum temperatures in the 9–35 °C range, and during the observation period, the number of days below each threshold was used as a variable; i.e. 27 variables in total were generated based on the daily maximum temperatures. The other thresholds were set at 1 °C intervals for the daily minimum temperatures in the 4–32 °C range, and the number of days above each threshold was used as a variable; i.e. 29 variables in total were generated based on the daily minimum temperatures. Finally, GLMs with binomial distributions were constructed using the abundance rate between *M. japonicus* and *M. banzai* in each survey area as a response variable. The same variables used for the correlation test were adopted as explanatory variables for the GLMs.

### Ethics approval

All authors have read, understood, and complied as applicable with the statement on “Ethical Responsibilities of Authors”.

## Results

### Sediment temperature

Sediment temperatures were observed in 12 intertidal flats in 11 survey areas over 335 days from October 2, 2019, to August 31, 2020. A total of 32,160 temperature values were obtained in each study area during the survey period; the mean, maximum, and minimum temperatures and the mean daily range during the survey period are shown in Table S1 in Supplementary Information 1. A summary of the silt and clay content and daily temperature characteristics of the intertidal flats for each survey area is shown in Table 3. The Friedman test showed significant differences among the survey areas for daily mean temperature, range, maximum temperature, and minimum temperature (χ^2^ = 1282.28–1686.67, *P* < 0.001). The lowest temperature was recorded in the Saba-gawa River (3.2 ℃), and the Saba-gawa River, the Naka-gawa River, and the Himiori-gawa River had the lowest daily minimum temperatures in the survey areas. The maximum temperature was recorded at the Tsuyazaki Inlet (35.6 ℃), but the daily mean and maximum temperatures in the Hitotsuse-gawa River, the Shinden-gawa River (H and L), the Tomouchi-gawa River, and the Suzuta-gawa River were significantly higher than those in the other areas. Although no significant differences were observed in the mean, maximum, and minimum daily temperatures between the two sites of the Shinden-gawa River (H and L sites) (Scheffe’s test; χ^2^ = 0.37–13.29, *P* > 0.05), the daily range was significantly higher in H than in L (Scheffe’s test; χ^2^ = 57.92, *P* < 0.001).

**Table 3:** Silt and clay content and median (min–max) of daily intertidal sediment temperature in each site during the survey period.

Site name	Silt & clay content (%)	Daily sediment temperature (°C)			
		Mean	Range	Maximum	Minimum
Naka-gawa Riv	48.4	17.5 (8.8–30.0)^d^	2.7 (0.2–6.9)^bc^	18.9 (9.9–31.8)^d^	16.0 (6.8–29.0)^d^
Satomi-gawa Riv	84.0	17.3 (7.9–32.0)^cd^	1.2 (0.2–4.0)^e^	18.1 (8.3–32.8)^d^	16.5 (7.3–31.4)^c^
Saba-gawa Riv	19.6	17.3 (6.2–32.2)^cd^	4.1 (0.8–10.1)^a^	19.2 (8.3–34.9)^bc^	14.9 (3.2–29.8)^d^
Himiori-gawa Riv	41.8	16.5 (7.7–32.3)^d^	2.6 (0.4–6.9)^c^	18.0 (8.8–33.5)^d^	15.3 (5.3–31.4)^d^
Tsuyazaki Inlet	21.0	17.6 (7.6–33.7)^b^	2.9 (0.4–8.9)^b^	19.5 (9.3–35.6)^b^	15.7 (5.1–32.0)^c^
Magome-gawa Riv	43.1	18.5 (10.3–31.0)^b^	2.7 (0.4–8.5)^bc^	19.9 (12.2–33.4)^b^	17.0 (7.2–29.7)^c^
Suzuta-gaaw Riv	56.6	19.6 (9.7–31.6)^a^	2.1 (0.1–7.5)^d^	21.1 (11.5–31.9)^a^	18.0 (7.5–31.4)^ab^
Imazato-gawa Riv	68.4	17.6 (9.1–30.9)^bc^	2.0 (0.3–5.3)^d^	19.0 (9.7–32.6)^cd^	16.3 (7.8–29.4)^c^
Tomouchi-gawa Riv	36.9	21.0 (13.9–28.4)^a^	2.2 (0.2–5.5)^d^	21.9 (14.5–30.7)^a^	19.7 (12.6–27.3)^ab^
Hitotsuse-gawa Riv	37.2	20.4 (13.7–31.2)^a^	2.7 (0.3–5.8)^bc^	21.4 (14.8–32.9)^a^	19.4 (11.7–30.1)^b^
Shinden-gawa Riv. (H)	28.3	19.9 (13.6–31.4)^a^	1.9 (0.4–5.9)^d^	21.1 (14.4–32.4)^a^	19.0 (11.4–30.5)^ab^
Shinden-gawa Riv. (L)	55.6	19.9 (14.1–30.7)^a^	1.5 (0.1–4.0)^e^	20.5 (14.7–32.0)^a^	19.1 (12.5–30.1)^a^

Sheffe’s test; a > b > c > d > e, *P* < 0.05.

**Figure 2:**
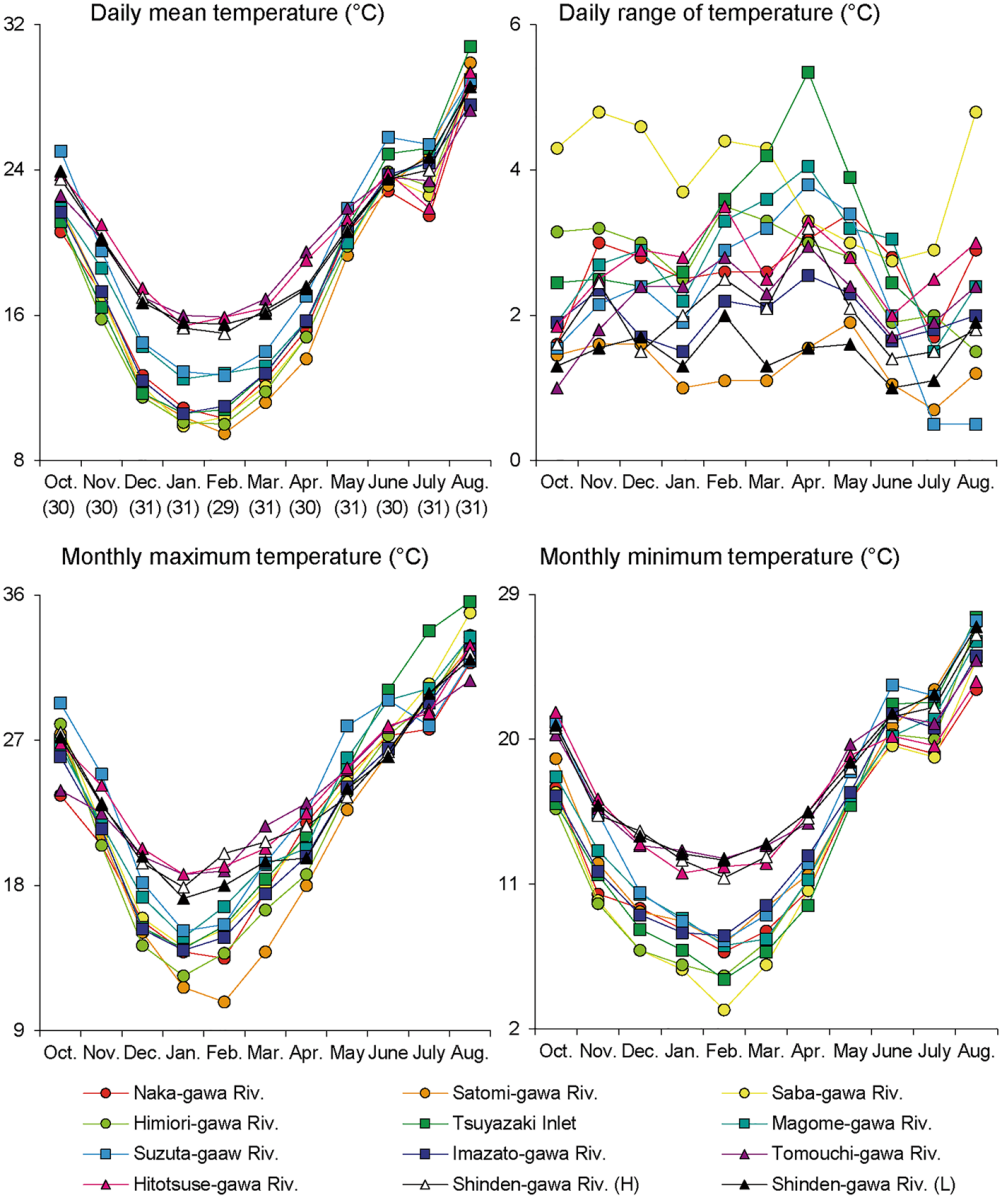
Daily mean intertidal sediment temperature, daily range, monthly maximum temperature, and monthly minimum temperature for the 12 survey areas during the observation period. The daily mean temperature and the daily range are indicated as the median values for each month.

The monthly temperature characteristics of the intertidal sediments are shown in Fig. 2. The daily mean, monthly maximum, and monthly minimum temperatures showed similar trends among the survey areas; however, the daily ranges varied. The temperatures gradually decreased from October, when the first observations were logged, and the minimum temperature was recorded in January at the Hitotsuse-gawa River and in February at the other 10 survey areas. After February, the temperature increased in each survey area, and August was the hottest month during the observation period in all areas. The largest differences in the daily mean, monthly maximum, and monthly minimum temperatures among the survey areas were recorded in February, with 6.4 °C (Min; 9.5 ℃ in the Satomi-gawa River, Max: 15.9 ℃ in the Tomouchi-gawa River and Hitotsuse-gawa River), 9.2 °C (Min; 10.8 ℃ in the Satomi-gawa River, Max; 20.0 ℃ in the Shinden-gawa River (H)), and 9.4 °C (Min; 3.2 ℃ in the Saba-gawa River, Max; 12.6℃ in the Tomouchi-gawa River), respectively.

**Figure 3:**
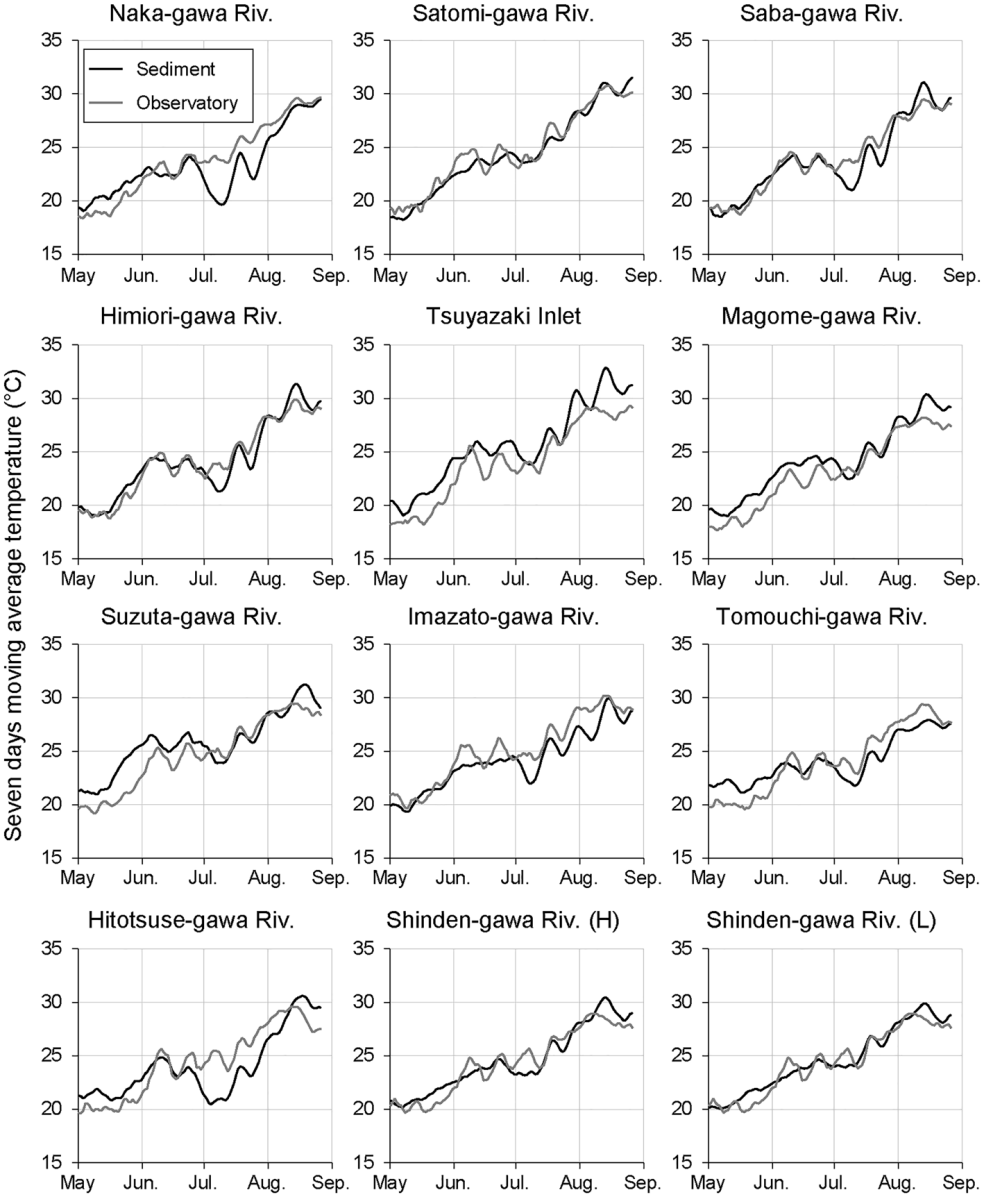
Seven-day moving averages of intertidal sediment and air temperatures during May–August 2020.

**Figure 4:**
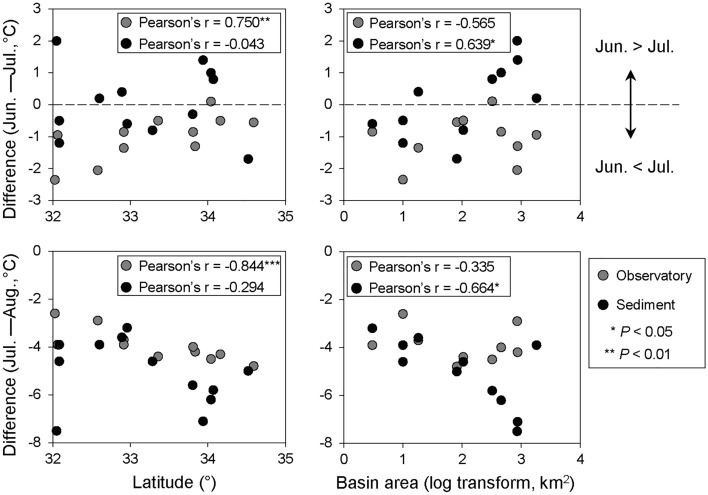
Relationship of intertidal sediment and air temperature differences with latitude and watershed area during June–July (upper two figures) and July–August 2019 (lower two figures). The median value for each month was used for estimating the temperature difference.

Although the sediment temperature showed an increasing trend from February 2019 to August 2020, it stagnated or decreased from June to July (Fig. 2). To assess the factors that contributed to this decrease in sediment temperature, we reevaluated the trends of 7-day moving averages of the intertidal sediment and air temperatures between May and August. The results suggested that the trends of intertidal sediment and air temperatures were generally synchronous seasonal variations in each survey area; however, a clear decrease in intertidal sediment temperatures was observed from the second half of June to the first half of July in several survey areas (e.g. the Naka-gawa and Hitotsuse-gawa rivers) (Fig. 3). The median values of the daily mean intertidal sediment and air temperatures were extracted for each survey area for each month from June to August, and the relationships between the difference in the temperatures between June and July and between July and August with latitude, longitude, silt and clay content, and basin area (log transformed) were analyzed. The difference in air temperature showed significant correlations with latitude only, whereas that in the intertidal sediment temperature showed significant correlations with basin area only (Fig. 4; Table S2 in Supplementary Information 1).

The correlations of the mean, maximum, and minimum intertidal sediment temperatures and the mean daily range during the observation period (335 days) with the spatial and environmental factors are shown in Table 4. The mean and minimum intertidal sediment temperatures showed significant positive correlations with the mean air temperature and significant negative correlations with latitude. The mean daily range was significantly negatively correlated with only silt and clay content values, and the maximum intertidal sediment temperature was negatively correlated with only the mean air temperature.

**Table 4:** Pearson’s correlation coefficient between intertidal sediment temperature and each variable.

	Sediment temperature (°C)			
	Mean	Daily range	Maximum	Minimum
Mean air temperature (°C)	0.807**	− 0.396	− 0.607*	0.787**
Mean dairy range of air temperature(°C)	− 0.159	0.128	0.248	− 0.160
Maximum air temperature (°C)	− 0.556	0.197	0.243	− 0.453
Minimum air temperature (°C)	0.364	− 0.276	− 0.380	0.327
Silt & clay content (%)	− 0.148	− 0.763**	− 0.454	0.129
Latitude (°)	− 0.915***	0.290	0.475	− 0.849***
Longitude (°)	− 0.465	− 0.066	− 0.188	− 0.155
Basin area (logit transformation, km^2^)	− 0.159	0.492	0.041	− 0.122

*P < 0.05, **P < 0.01, ***P < 0.001.

Tsuyazaki inlet is not included in the basin; therefore, the sample size for *Basin area* was 11.

**Figure 5:**
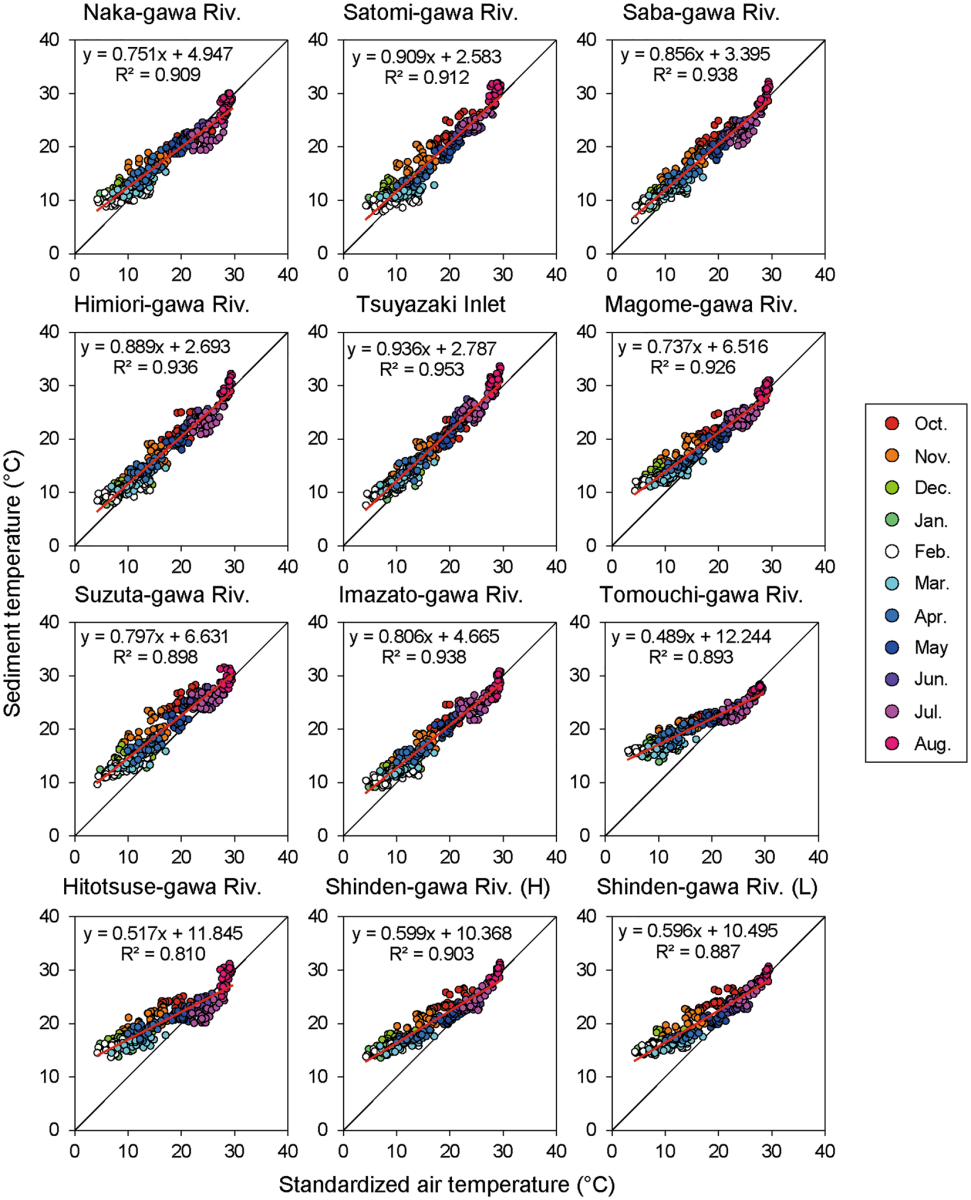
Relationship between mean daily intertidal sediment temperature and standardized air temperature in each survey area during the survey period.

The daily mean of 335 days of intertidal sediment temperatures and standardized air temperatures were positively correlated in each survey area (Fig. 5). With 207–304 of the 335 days of the plots located on the upper diagonal, the intertidal sediment temperature was higher than the air temperature for more than 60% of the days during the observation period. In the winter season between December 2019 and February 2020, a gap between the intertidal sediment and standardized air temperatures was observed, and the former was higher than the latter by more than 10 °C in several survey areas (Fig. S1 in Supplementary Information 2). In contrast, between June and August, the standardized temperature was higher than the intertidal sediment temperature on several days (Fig. 5; Fig. S1 in Supplementary Information 2).

### Relationship between sentinel crab species and sediment temperature

**Figure 6:**
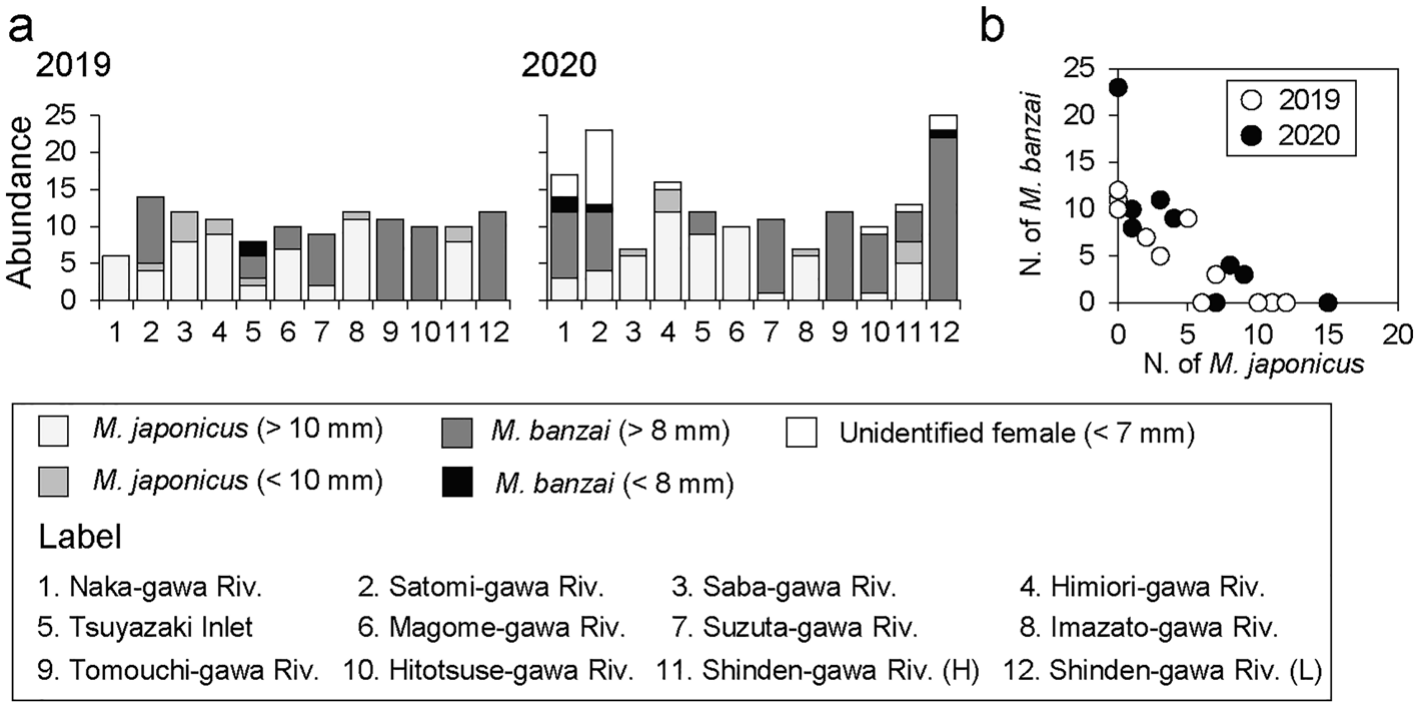
Numbers of *Macrophthalmus japonicus* and *Macrophthalumus banzai* collected at each survey area in 2019 and 2020 (**a**), and relationship between the two species abundances (**b**).

Sixty-eight and 57 individuals of *M. japonicus* and *M. banzai* were collected in 2019, respectively, and 65 and 80 individuals were collected in 2020, respectively (Fig. 6a). The minimum CW of the ovigerous females was 16.8 mm for *M. japonicus* and 8.2 mm for *M. banzai* (Fig. S2 in Supplementary Information 2). The number of overwintering *M. japonicus* individuals was 57 in 2019 and 2020, and that of overwintering *M. banzai* was 55 and 76 in 2019 and 2020, respectively. *M. japonicus* was not collected from the Tomouchi-gawa and Shinden-gawa rivers (L), whereas *M. banzai* was not collected from the Saba-gawa, Himiori-gawa, and Imazato-gawa rivers. The numbers of the two species at each survey area in both 2019 and 2020 showed significant negative correlations (Spearman’s r_s_ in 2019; − 0.899, *P* < 0.01, r_s_ in 2020; − 0.759, *P* < 0.01) (Fig. 6b).

**Figure 7:**
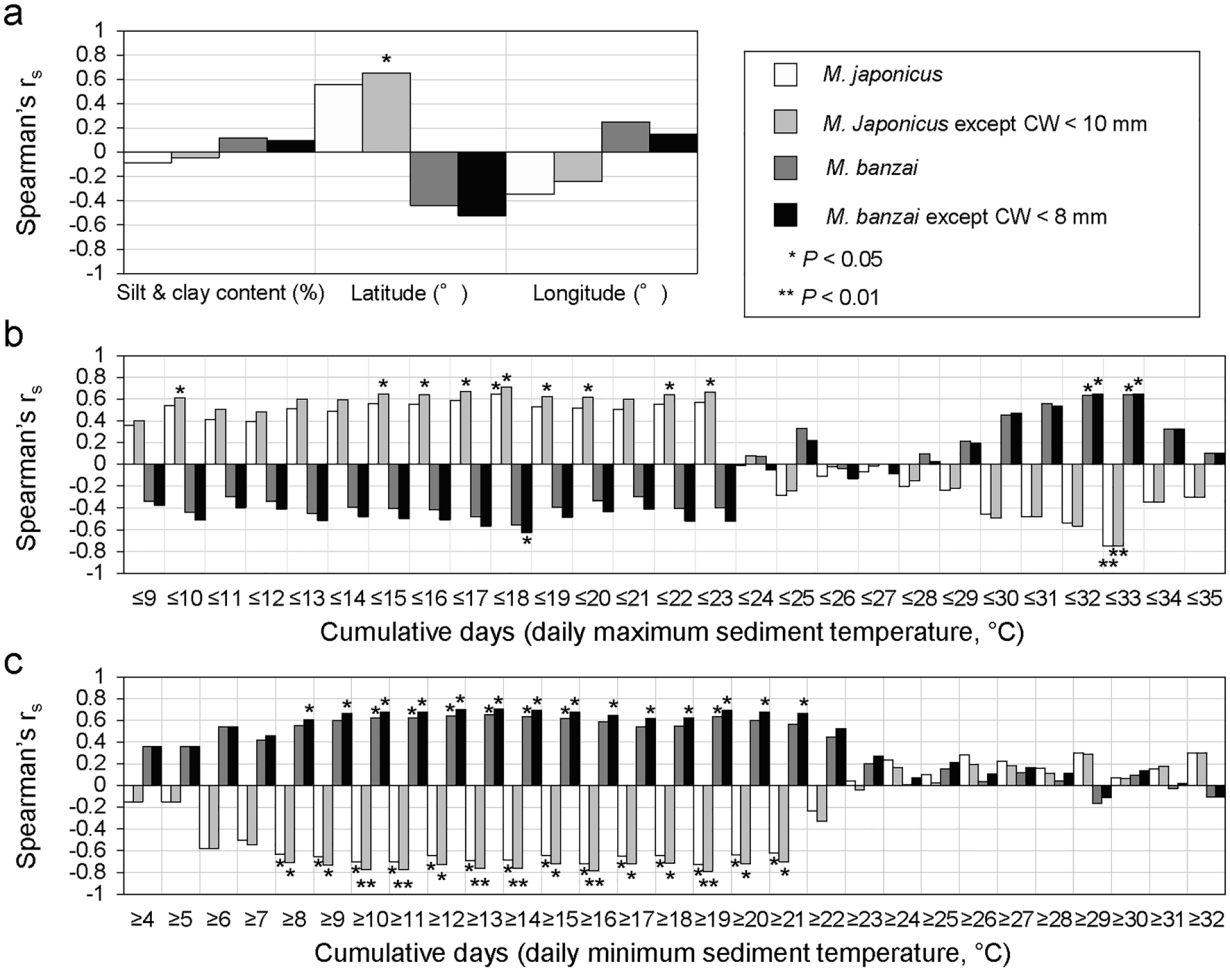
Spearman’s rank correlation coefficient between the number of *Macrophthalmus japonicus* and *Macrophthalumus banzai* and 59 variables. (**a**) indicates the coefficients for three variables (silt and clay content, latitude and longitude), (**b**) for the 27 variables based on the daily maximum sediment temperatures, and (**c**) for 29 variables based on the daily minimum temperatures.

The correlation coefficients between the numbers of *M. japonicus* and *M .banzai* and their overwintering individuals, and 59 variables are shown in Fig. 7. The number of *M. japonicus* was significantly correlated with 16 variables and correlated most strongly with the number of days with a daily maximum temperature ≤ 33 °C (r_s_ =  − 0.750). The number of overwintering *M. japonicus* showed significant correlations with 25 variables and the strongest correlation with the number of days with a daily minimum temperature ≥ 19 °C (r_s_ =  − 0.790). The number of *M. banzai* was significantly correlated with nine variables and most strongly correlated with the number of days with a daily minimum temperature ≥ 13 °C (r_s_ = 0.653). The number of overwintering *M. banzai* was significantly correlated with 17 variables, and was most strongly correlated with the number of days with a daily minimum temperature ≥ 13 °C (r_s_ = 0.706).

**Figure 8:**
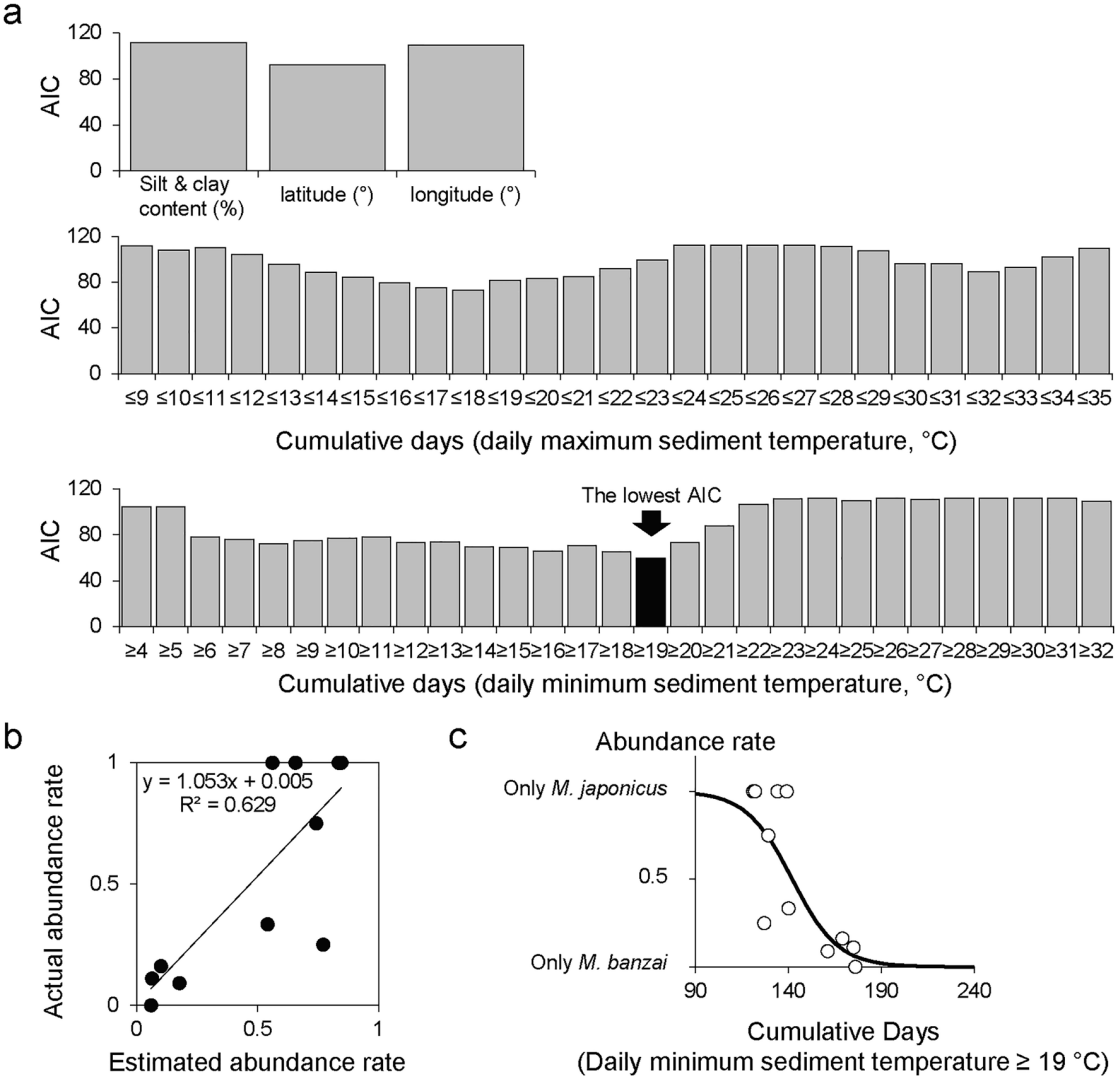
Akaike information criteria (AIC) of the generalized linear model constructed for each of the 59 explanatory variables (**a**), the relationship between the actual and estimated values (i.e. abundance rate of *Macrophthalmus japonicus* and *Macrophthalumus banzai*) of the model (**b**), and the response curve based on the variable with the lowest AIC (**c**).

Using each of the 59 variables, GLMs were constructed to estimate the proportions of overwintering *M. japonicus* and *M. banzai*. The results showed that the number of days with daily minimum temperature ≥ 19 °C had the lowest Akaike’s information criterion (AIC) (Fig. 8a); consequently, the model using this variable was regarded as the best model. The actual abundance rate and the abundance rate estimated by the best model were significantly positively correlated (Pearson’s r = 0.793, *P* < 0.01) (Fig. 8b), confirming a certain level of prediction accuracy. According to the response curve based on the best model (Fig. 8c), the abundance rate of *M. banzai* exceeded 50% and became dominant when the number of days with a daily minimum temperature ≥ 19 °C exceeded 142 days. In most of the survey areas dominated by *M. japonicus*, the minimum daily temperature was below 19 °C from late October 2019 to the middle of May 2020, whereas in the survey areas dominated by *M. banzai*, the minimum daily temperature was below 19 °C from the middle of November 2019 to early May 2020 (Fig. S3 in Supplementary Information 2).

## Discussion

### Characteristics of sediment temperature

Although several previous studies have evaluated the sediment temperatures of intertidal flats^9,11–13^, to the best of our knowledge, few studies have investigated the differences in sediment temperature characteristics among several intertidal flats in various regions [e.g. Ref.^31^]. In the present study, intertidal sediment temperatures were observed in 12 intertidal flats in 11 survey areas inhabited by the sentinel crab species *M. japonicus* and/or *M. banzai* over a period of approximately one year. The intertidal sediment temperature differed among the regions (Table 3) and the mean and minimum temperatures during the survey period showed significant negative correlations with latitude and significant positive correlations with mean air temperature (Table 4). In contrast, the maximum temperature during the observation period exhibited a significant negative correlation with the mean air temperature (Table 4), and the gap between the maximum and minimum temperatures during the survey period was smaller in the survey area at lower latitudes (Fig. 5). Cho et al.^12^ demonstrated the influence of insolation on intertidal sediment temperature. Generally, insolation is higher in lower latitudinal regions than in higher latitudinal regions, resulting in a latitudinal gradient of air and surface marine temperatures^32^. Therefore, the intertidal sediment temperature is considered to be affected by factors that respond to latitudinal gradients, as well as by air and surface marine temperatures.

Our data support the idea that intertidal sediment temperatures are also influenced by basin characteristics and that this influence is particularly pronounced during the summer months. In several rivers, a decrease in intertidal sediment temperature was observed from June to July (Figs. 2 and 3). Saravanakumar et al.^33^ also observed a similar phenomenon in the sediments of mangrove forests in India, and considered that the monsoon influenced it. Moreover, intertidal sediment temperatures were higher than the normalized air temperature for more than 60% of the days during the observation period. However, from June to August, the normalized air temperature was higher than the intertidal sediment temperature across relatively more number of days (Fig. 5 and Fig. S1 in Supplementary Information 2). In Japan, rainfall increases and insolation decreases during this period because of the monsoon (Japan Meteorological Agency: https://www.data.jma.go.jp/gmd/cpd/longfcst/en/tourist_japan.html). In other words, the seasonal decrease in insolation and the increase in river water and groundwater due to rainfall are considered to have influenced the decrease in intertidal sediment temperature in early summer. Inland groundwater supply is one of the factors that contributes to the stabilization of intertidal sediment temperature^13^. The significant correlation between the basin area and intertidal sediment temperature (Fig. 4) suggests that the temperature in intertidal flats located in large basins is more strongly influenced by inland water.

In addition to the latitudinal gradient and basin characteristics, habitat-scale conditions, that is, intertidal flat characteristics, possibly affect the trend of intertidal sediment temperature in western Japan. Because the daily range of the Shinden-gawa River (H) was significantly higher than that of the L site (Table 3), it is reasonable to assume that factors related to ground elevation (i.e. flooding and solar radiation time) affected the daily range of intertidal sediment temperature, as reported by Cho et al.^12^. In the present study, the mean daily range of the observation period showed a significant and strong negative correlation with the silt and clay content (Table 4). Lim et al.^31^ showed that the thermal diffusivity was higher in sandy flats than in muddy flats. Our results and those of a previous study suggest that intertidal flats covered with fine sediments are less affected by thermal factors propagated from the sediment surface.

### Relationship between distribution of sentinel crab species and sediment temperature

The coexistence of *M. japonicus* and *M. banzai* was observed in seven of the 11 survey areas in the present study (Fig. 6a) and the numbers of the two species in each survey area showed a significant negative correlation (Fig. 6b). This suggests that they have an interspecific relationship in the tidal flats where they coexist. For example, competition for space and food resources can be expected because both species burrow in tidal flats and feed on the surface sediments^16,18,22,34^. However, our study was not intended to elucidate the habitat niche of the two sentinel crab species but to reveal the relationship between sediment temperature and distribution patterns of the two species. Our results are insufficient to comprehensively analyze the interspecific interactions between the two species, and thus, further experiments are required.

The abundance of each sentinel crab species in the survey areas was related to the intertidal sediment temperature (Fig. 7). The number of overwintering *M. banzai* was most strongly and positively correlated with the number of days with a daily minimum temperature ≥ 13 °C (r_s_ = 0.706, Fig. 7). Henmi^34^ reported that the feeding behavior of this species decreases in the intertidal sediment surface when the temperature falls below 15 °C, and many individuals remain in their burrows. Therefore, it is possible that the survival of *M. banzai* may decline during overwintering with increasing number of days (or longer periods) with the intertidal sediment temperature below 13 °C.

In contrast, the number of overwintering *M. japonicus* was most strongly and negatively correlated with the number of days with a daily minimum temperature ≥ 19 °C (r_s_ =  − 0.790, Fig. 7), and no relationship was found between winter temperatures and the abundance of this species. The overwintering *M. japonicus* showed a significant positive correlation with latitude (r_s_ = 0.653; Fig. 7), suggesting that the potential population may be smaller in regions with lower latitudes than in those with higher latitudes. In fact, there are concerns about the decline and local extinction of the species on the Tanegashima Island, which is the southern limit of its distribution in Japan, owing to the deterioration of its habitat caused by anthropogenic impacts^35^. However, Matsumasa et al.^36^ reported that the distribution range of the species had expanded to the northern region of Japan in recent years and suggested the possibility that the increase in winter temperatures due to climate change has contributed to its successful distributional expansion. Therefore, based on the results of the present study, we cannot completely deny that winter temperature are irrelevant to changes in the distribution range of this species. In other words, our hypothesis that spatial distribution patterns of these two species are closely related to the intertidal sediment temperature during the winter season was partially supported.

### Importance of ecological assessment of sentinel crabs and sediment temperature

Intertidal sediment temperatures have not been monitored well, despite the fact that intertidal sediment temperatures and their seasonal changes affect primary productivity and the distribution and abundance of benthos^15,37,38^. As discussed earlier, our results suggest that the sediment temperature in intertidal flats is influenced and characterized by a variety of spatial-scale factors. Thus, intertidal sediment temperature should be monitored, and long-term trends should be evaluated while considering the linkages with terrestrial and marine areas and the physical environmental conditions of intertidal flats.

Populations of *M. japonicus* and *M. banzai* may respond to future changes in the sediment temperature of the intertidal flats based on our findings. Our GLMs showed that in the survey areas, i.e. in the western Japan region where both species coexist, the abundance rates of the two species were not related to the latitudinal gradient, but were most related to the number of days with the daily minimum temperature ≥ 19 °C (Fig. 8). As shown earlier, winter temperatures were related to *M. banzai* abundance. In the future, the temperature in Japan has been predicted to increase relatively more in winter than in summer^39^, which may lead to an increase in the population of *M. banzai* in various regions and a corresponding decrease in the population of *M. japonicus*. These two species have coexisted in western Japan since at least the 1970s^21,22^, but the fluctuations in the populations of the two species over the past half-century have not been fully evaluated. As species replacement associated with climate change may alter intertidal ecosystems, it is necessary to monitor population changes in the future.

Nevertheless, southern species are not always replaced by northern species because of climate change [e.g. Ref.^40^]. Our model shows that the increase in the number of days with temperature ≥ 19 °C (i.e. the increase in intertidal sediment temperatures from late fall to late spring, Fig. S3 in Supplementary Information 2) caused changes in the abundance rate of *M. japonicus*/*M. banzai*; however, the temperature tolerance of each species was not evaluated in the present study. The temperature tolerance of the target species must be evaluated to understand the expansion and/or contraction of its distribution range^27,41^. Further research is required to address these gaps regarding the ecological traits of the two species.

## Conclusion

We found that the sediment temperature of intertidal flats is influenced by various spatial scales such as latitudinal, basin, and habitat scales. Furthermore, our findings indicate that intertidal sediment temperature may affect the distribution pattern of the two sentinel crab species in the intertidal flats where they coexist. Although the distribution pattern of *M. banzai* was related to winter temperature, that of *M. japonicus* was not; thus, our hypothesis was partially supported. Our findings emphasize the importance of long-term monitoring of sediment temperature and sentinel crab species in intertidal flats to evaluate the influence of future climate change.

### Supplementary Information

Supplementary Tables.

Supplementary Figures.

## Data Availability

The datasets obtained and/or analyzed by the authors’ fieldwork during the present study are available from the corresponding author on reasonable request.
