# Supplementary material for: Sediment temperature characteristics and their relation to distribution patterns of two sentinel crab species in intertidal flats in western Japan
Source: Sci Rep. 2024 Jan 9;14:861. doi: 10.1038/s41598-024-51515-8 (PMC10776695; doi:10.1038/s41598-024-51515-8)
Supplement: Supplementary file 1 — Supplementary Tables. [file 41598_2024_51515_MOESM1_ESM.pdf]

Table S1. Mean, maximum, minimum temperature and mean daily range during the survey period (335 days, the number of logging data = 32,160) in each survey area.

Survey Area	Sediment temperature (°C)			
	Mean ± Standard Deviation (SD)	Mean daily range ± SD	Maximum	Minimum
Naka-gawa Riv.	17.5 ± 5.6	2.8 ± 1.2	31.8	6.8
Satomi-gawa Riv.	17.8 ± 6.7	1.3 ± 0.7	32.8	7.3
Saba-gawa Riv.	17.7 ± 6.4	4.0 ± 1.7	34.9	3.2
Himiori-gawa Riv.	17.6 ± 6.5	2.7 ± 1.2	33.5	5.3
Tsuyazaki Inlet	18.4 ± 6.9	3.2 ± 1.6	35.6	5.1
Magome-gawa Riv.	18.8 ± 5.5	2.9 ± 1.6	33.4	7.2
Suzuta-gaaw Riv.	20.0 ± 6.0	2.3 ± 1.5	31.9	7.5
Imazato-gawa Riv.	18.1 ± 5.9	2.2 ± 1.1	32.6	7.8
Tomouchi-gawa Riv.	20.4 ± 3.7	2.3 ± 1.2	30.7	12.6
Hitotsuse-gawa Riv.	20.5 ± 4.1	2.7 ± 1.2	32.9	11.7
Shinden-gawa Riv. (H)	20.4 ± 4.5	2.1 ± 1.0	32.4	11.4
Shinden-gawa Riv. (L)	20.5 ± 4.5	1.6 ± 0.8	32.0	12.5

Table S2. Pearson's correlation coefficients between the difference in air/intertidal sediment temperature and spatial and environmental factors.

Variable name	Air temperature (°C)		Sediment temperature (°C)	
	Jun.–Jul.	Jul. –Aug.	Jun.–Jul.	Jul. –Aug.
Latitude in observatory (°)	0.750**	−0.844**		
Longitude in observatory (°)	0.155	−0.406		
Latitude in survey site (°)			−0.043	−0.294
Longitude in survey site (°)			0.263	−0.560
Basin area (log trans., km^2)	0.053	−0.075	0.639*	−0.664*
Silt & clay content (%)	0.072	−0.224	−0.498	0.394

* $P < 0.05$, ** $P < 0.01$

Tsuyazaki Inlet is not included in the basin; therefore, the sample size for *Basin area* was 11.
