# Supplementary material for: Sediment temperature characteristics and their relation to distribution patterns of two sentinel crab species in intertidal flats in western Japan
Source: Sci Rep. 2024 Jan 9;14:861. doi: 10.1038/s41598-024-51515-8 (PMC10776695; doi:10.1038/s41598-024-51515-8)
Supplement: Supplementary file 2 — Supplementary Figures. [file 41598_2024_51515_MOESM2_ESM.pdf]

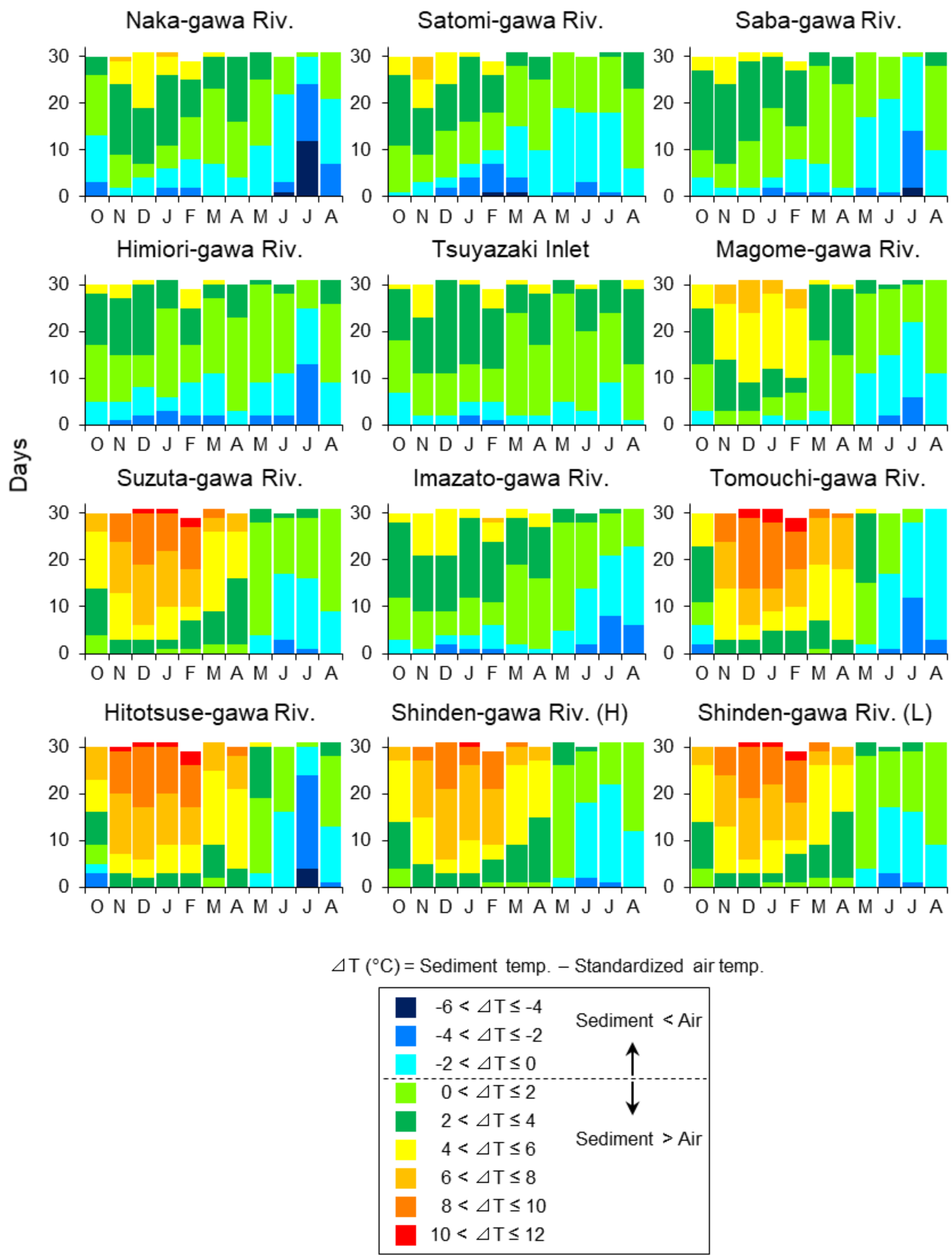

Fig. S1. Differences between intertidal sediment temperature and standardized air temperature (ΔT) during the survey period between October 2, 2019 and August 31, 2020 (335 days). ΔT of each day is classified into nine levels according to its value and organized by month.

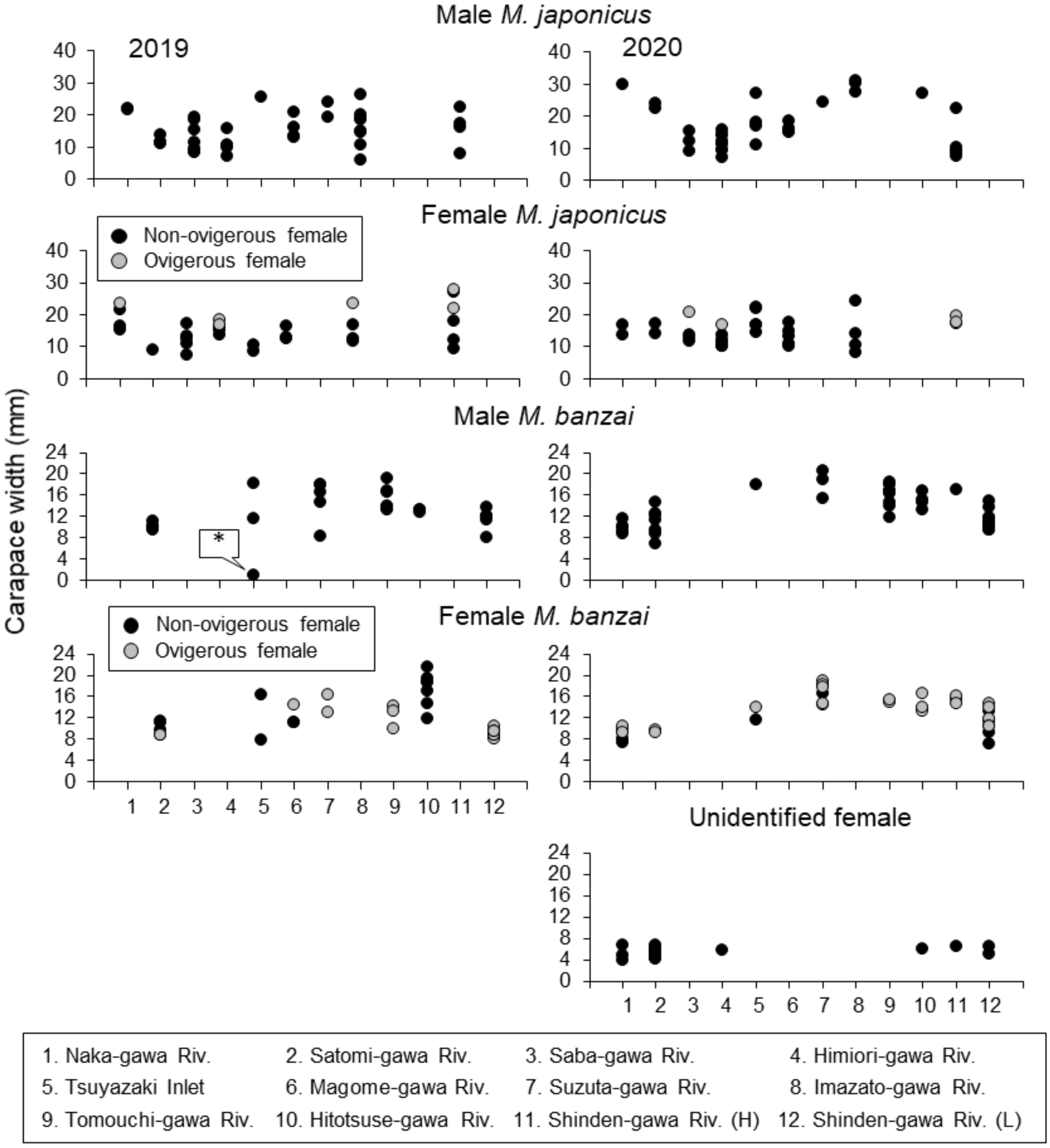

Fig. S2. Carapace width (CW) of male and female *Macrophthalmus japonicus* and *Macrophthalmus banzai* at each survey area in 2019 and 2020. An asterisk (*) indicates the individual whose carapace was damaged and its CW could not be measured.

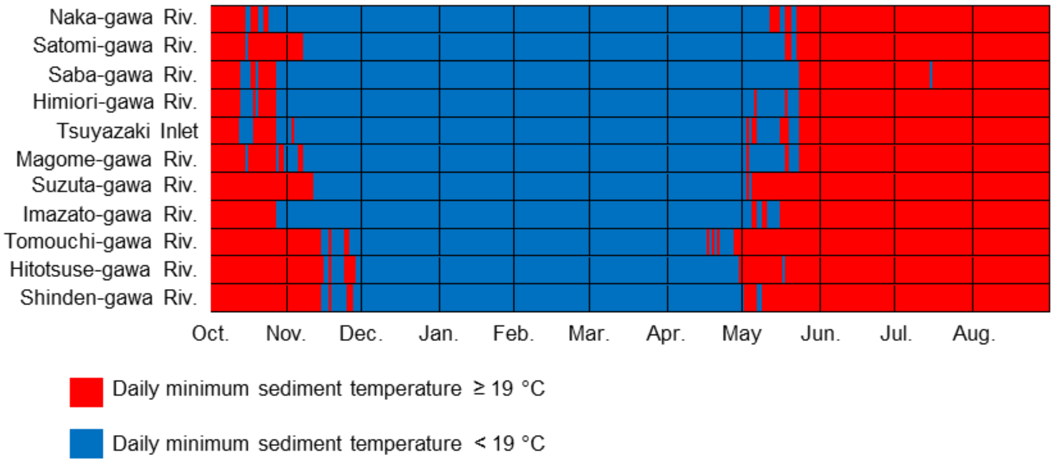

Fig. S3. Days with a daily minimum temperature of 19 °C as the threshold, at intertidal flats in each survey area during the survey period: between October 2, 2019 to August 31, 2020 (335 days).
